# Elucidating the evolution of litchi: insights into the population genomics of ancient trees on origin, dispersal, and adaptive genetic basis

**DOI:** 10.1093/hr/uhag226

**Published:** 2026-07-13

**Authors:** Han Wang, Xiaoqian Yu, Lianlian Rao, Mianqiao Pan, Zuanxian Su, Houbin Chen

**Affiliations:** Guangdong Litchi Engineering Research Center, College of Horticulture, South China Agricultural University, Guangzhou 510642, China; Guangdong Laboratory of Lingnan Modern Agriculture, South China Agricultural University, Guangzhou 510642, China; Guangdong Litchi Engineering Research Center, College of Horticulture, South China Agricultural University, Guangzhou 510642, China; Guangdong Laboratory of Lingnan Modern Agriculture, South China Agricultural University, Guangzhou 510642, China; Guangdong Litchi Engineering Research Center, College of Horticulture, South China Agricultural University, Guangzhou 510642, China; Guangdong Laboratory of Lingnan Modern Agriculture, South China Agricultural University, Guangzhou 510642, China; Guangdong Litchi Engineering Research Center, College of Horticulture, South China Agricultural University, Guangzhou 510642, China; Guangdong Laboratory of Lingnan Modern Agriculture, South China Agricultural University, Guangzhou 510642, China; Guangdong Litchi Engineering Research Center, College of Horticulture, South China Agricultural University, Guangzhou 510642, China; Guangdong Laboratory of Lingnan Modern Agriculture, South China Agricultural University, Guangzhou 510642, China; Guangdong Litchi Engineering Research Center, College of Horticulture, South China Agricultural University, Guangzhou 510642, China; Guangdong Laboratory of Lingnan Modern Agriculture, South China Agricultural University, Guangzhou 510642, China

## Abstract

Litchi (*Litchi chinensis* Sonn.) is a high-value subtropical fruit tree native to eastern Yunnan, China. Its cultivation was recorded in the second century BC. Ancient litchi trees are widespread across southern China, some over 1000 years old, yet their genetic potential remains largely uninvestigated. Here, we analyzed resequencing data from 93 *in situ* ancient litchi trees with integrated public datasets and simulated paleoclimate species distribution models (SDMs) using over 20 000 ancient litchi distribution records. This approach led to several significant findings. First, population analyses identified two wild lineages (Yunnan, Hainan) and five admixed clusters. SDMs indicate last glacial maximum contraction with a Hainan refugium, followed by postglacial expansion across South China while Yunnan lagged. Principal component analysis and runs of homozygosity further revealed fine-scale population structure. Second, several historical dispersal events of litchi were uncovered. Most ancient Sichuan trees were derived from the Yunnan wild lineage, with fragmented montane habitats supporting human-mediated introduction. Ancient Fujian and Guangdong trees are chiefly admixed cultivars, with genetic distances indicating clonal spread across regions. Third, we identified signals where variation in flowering time and disease resistance coincides with highly heterozygous genomic blocks and haplotype-biased transcription. This pattern is observed in flowering differences between cultivars ‘Feizixiao’ and ‘Nuomici’ and in disease responses between the ancient resistant cultivar ‘Heiye’ and the susceptible cultivar ‘Guiwei’. Our research provides a genomic resource for ancient litchi trees, offering new insights into litchi dispersal, cultivation, and priorities for breeding.

## Introduction

The litchi (*Litchi chinensis* Sonn.), a crucial economic subtropical fruit, originated in Yunnan, China [1]. Historical chronicles and archaeological findings in the Hepu Han Tomb (Guangxi, China) suggest a cultivation history spanning over 2000 years [2]. Litchi trees also feature the longest productive lifespan among tropical and subtropical fruits. The age of the well-known ‘Songxiang’ litchi tree in Fujian, China, has surpassed ~1250 years [1]. An unpublished survey reveals that there are more than 19 000 litchi trees aged over 100 years in Maoming city, Guangdong, China. Among these ancient trees, the cultivar ‘Heiye’ is the most predominant, accounting for 60% of the total. Radiocarbon dating has demonstrated that the oldest tree is around 1900 years old. Notably, almost all of these ancient litchi trees remain productive in fruit bearing.

Ancient trees serve a pivotal function in disclosing the domestication and cultivation histories of diverse plant species. For instance, in the ‘Taula del Sénia’ region, the cultivar ‘Farga’ was preferred by historical agriculturists, comprising 97% of ancient olive trees, as shown by an analysis using eight SSR markers [3]. Moreover, an analysis of chloroplast DNA haplotypes implies that these trees were either cross-bred with wild olive resources or directly introduced [3]. Similarly, investigations of ancient trees substantially augment our comprehension of genetic diversity, phylogenetic associations, domestication processes, and longevity in species such as poplar [4], tea [5], ginkgo [6], and oak [7].

Regional investigations of ancient litchi germplasm in Guangxi, Fujian, and the lower reaches of the Minjiang River, employing ISSR, RAPD, and SRAP markers, consistently indicate high polymorphism and extensive genetic diversity [8–11]. Nevertheless, the methodological and sampling limitations intrinsic to these studies restrict their resolution. Hence, a cross-regional whole-genome resequencing survey of ancient litchi is requisite. Whole-genome resequencing has emerged as a fundamental pillar in plant genomics, facilitating the exploration of genetic diversity, population structure, and trait architecture in economically significant crops. The publication of a high-quality genome of litchi [1] established a crucial basis for population-scale genomic investigations. Building upon this foundation, large-scale resequencing endeavors have uncovered both the population structure and the genetic underpinnings of fruit quality traits, including the discovery of soluble acid invertase (*LcSAI*), an invertase gene that governs sugar composition and sweetness [12]. The genotyping resources have also been expanded with the development of a versatile liquid single nucleotide polymorphism (SNP) chip derived from 875 deeply sequenced accessions, which enables high-resolution mapping, germplasm digitalization, and the identification of agronomic trait loci [13]. Collectively, these genomic resources have enhanced our comprehension of modern cultivars; however, the genetic background of ancient litchi trees remains inadequately characterized.

In tandem with the progress in genomics, species distribution modeling (SDM) has proven valuable for reconstructing population histories in response to Quaternary climate shifts. SDM for ginkgo revealed significant habitat contraction during the Last Glacial Maximum (LGM), followed by postglacial expansion, highlighting distinct refugial dynamics across China [14]. Analogous analyses conducted on *Populus cathayana* revealed glacial refugia and severe bottlenecks associated with Quaternary climate changes [15]. Meanwhile, research on *Engelhardia roxburghiana* reconstructed population contractions and expansions throughout the LGM, which showed a strong correspondence with the demographic histories deduced from genomic data [16]. These studies emphasize the efficacy of integrating SDM with population genomics to elucidate historical dispersal, refugial persistence, and demographic trajectories.

This study generates a comprehensive whole-genome variation dataset for litchi, based on field investigations that sampled 93 ancient trees from the provinces of Fujian, Sichuan, Yunnan, and Guangdong in China. Phylogenetic, population structure, and principal component analyses (PCA) based on various types of SNPs, along with runs of homozygosity (ROH) and genetic distance calculations, were performed to explore litchi’s genetic background and historical dispersal. SDM complements these genomic analyses, offering insights into how past climate fluctuations affected litchi’s distribution and evolution. Additionally, analyses of high-heterozygosity regions and allele-specific expression (ASE) identified key genes related to flowering and disease resistance. These findings deepen our understanding of litchi domestication, cultivation history, and provide essential genomic resources for the conservation and further study of ancient litchi trees.

## Results

### Whole-genome sequencing of ancient litchi samples

A total of 107 litchi samples were collected, among which 93 were from ancient trees estimated to be aged between 100 and 1900 years. These ancient trees, collected from 29 different locations, are mainly preserved *in situ*, either on farms or in temples (Table S1). All 107 samples were subjected to 150-bp paired-end whole-genome sequencing, attaining an average sequencing depth of ~25× and an average mapping coverage of 94.50% against the ‘Feizixiao’ (‘FZX’) reference genome (Fig. 1B; Table S1). To improve genomic resolution, resequencing data from previously published litchi accessions [1] were integrated. A total of 29 789 611 high-quality biallelic SNPs and 3 082 555 small insertions and deletions (InDels) were identified from 175 samples (Fig. 1B; Table S2). The majority of adjacent SNPs had a spacing of less than 50 bp. Among the identified SNPs, 13 921 869 (46.73%) were low-frequency variants with a minor allele frequency (MAF) < 5%. Of the common SNPs (MAF ≥ 5%), 884 036 (5.57%) were situated within coding sequences (CDS). In total, 504 876 nonsynonymous SNPs were detected across 28 122 genes, with a nonsynonymous to synonymous substitution ratio of 1.39 (Fig. 1C; Table S3).

**Figure 1:**
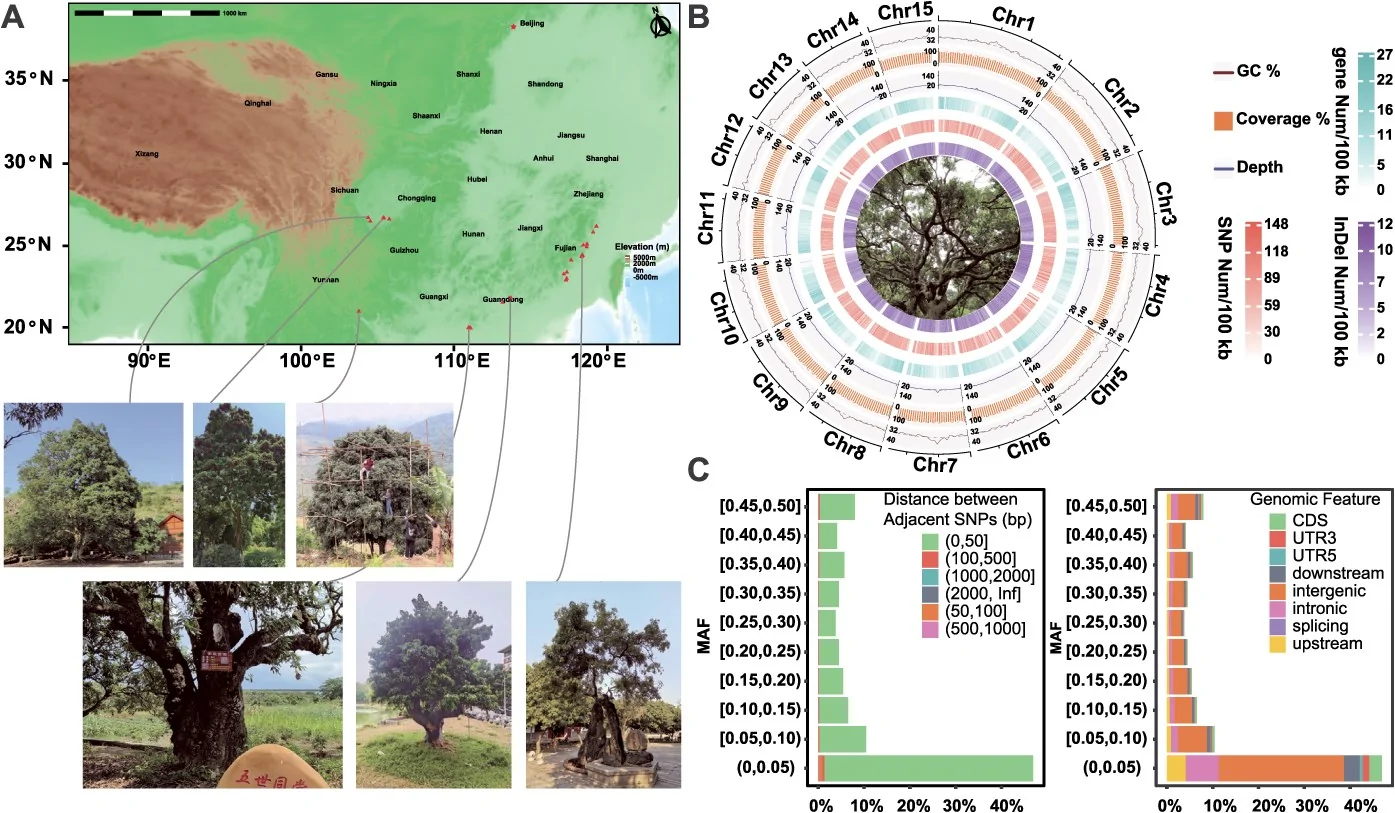
Whole-genome sequencing data for selected ancient litchi plants and their geographic origins. (A) The sampling locations of 93 ancient litchi trees. (B) Circos plots illustrating the various types of variants found in the 175 litchi samples at a genome-wide level. The tracks, arranged from outermost to innermost, represent the chromosome lengths of the reference genome, the GC content of the reference genome, the average coverage of sequencing samples, the average depth of sequencing samples, the gene density of the reference genome, and the density of SNPs and small insertions and deletions (InDels). Variant density was calculated using nonoverlapping 100-kilobase window intervals. (C) The distribution of MAF, adjacent SNP distance, and function annotation for SNPs identified from ancient litchi populations. SNPs are grouped according to the distance between neighboring SNPs or their putative functional categories.

### In-depth analysis of the population structure of litchi using various groups of SNPs

The phylogenetic tree classified all litchi accessions into seven distinct genetic clusters, which were composed of two major groups (Cluster 1 and Cluster 2) and five intermediate clusters (H1–H5) (Fig. 2A). Cluster 1 encompassed the majority of ancient litchi accessions originating from Sichuan Province, wild accessions from Yunnan Province (YNW), Vietnam, and Guangxi Province, along with extremely early-maturing cultivars (EEMCs). Cluster 2 consisted of wild resources from Hainan Province (HNW), late-maturing cultivars (LMCs), and several ancient accessions from Fujian and Guangdong Provinces.

**Figure 2:**
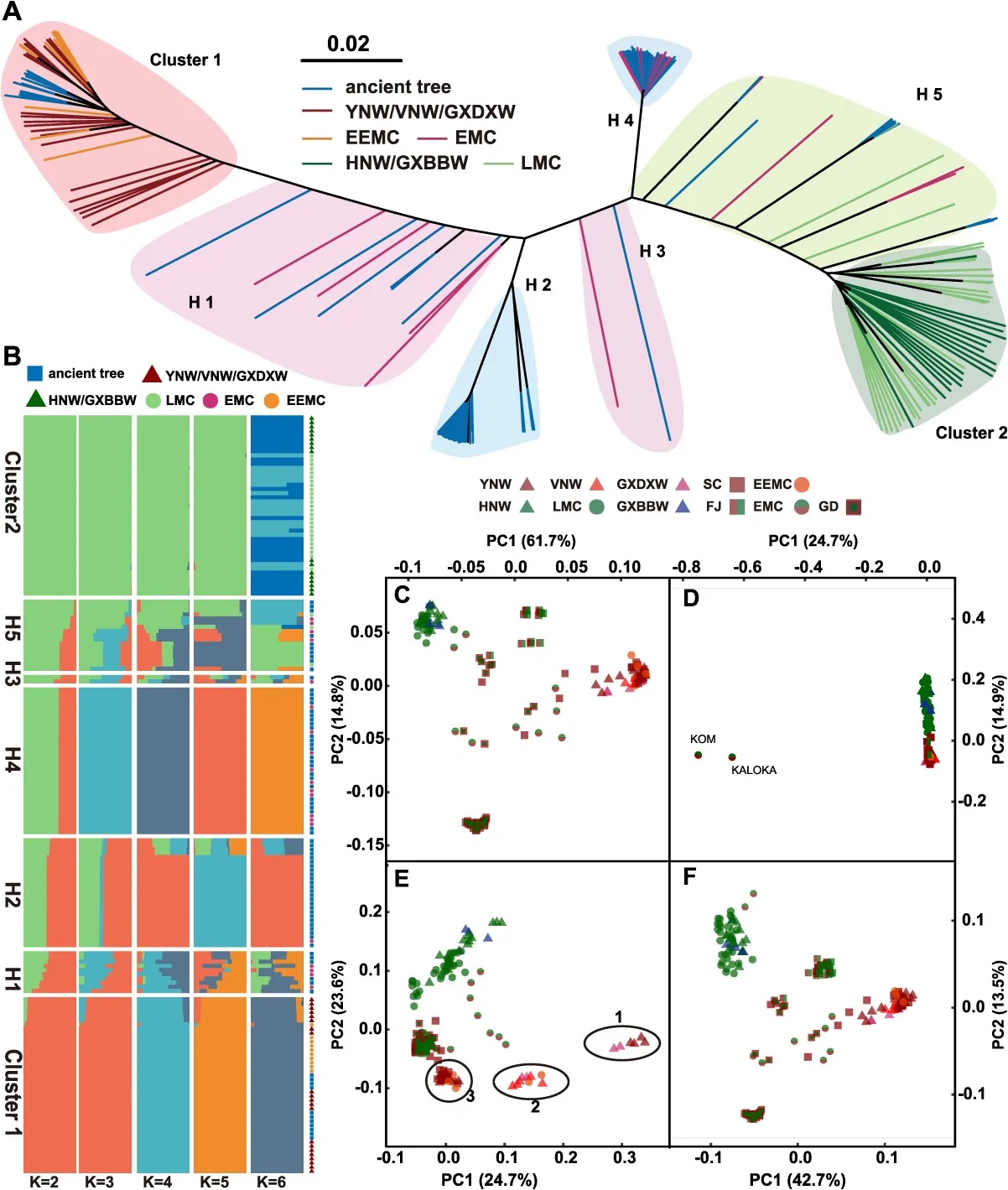
Population genomic structure of 175 litchi accessions. (A) Neighbor-joining (NJ) phylogenetic tree constructed using high-quality whole-genome SNPs. Branch colors represent distinct groups: blue, ancient litchi trees; red, wild accessions from Yunnan and adjacent regions (including Yunnan [YNW], Vietnam [VNW], and Daxin, Guangxi [GXDXW] wilds); orange, EEMC; pink, EMC; dark green, wild accessions from Hainan and adjacent regions (including Hainan [HNW] and Bobai, Guangxi [GXBBW] wilds); and light green, LMC. (B) Estimated population structure by ADMIXTURE analysis for *K* = 2–6. The *y*-axis shows populations, and the *x*-axis quantifies the proportions of inferred ancestral lineages. The legend on the right denotes sample categories: squares (ancient trees), triangles (wild accessions), and circles (cultivars). (C–F) PCA of 175 litchi accessions based on the following SNP subsets. (C) common SNPs (MAF ≥ 0.05). (D) rare SNPs (MAF < 0.05); (E) highly heterozygous SNPs (observed heterozygosity > 0.8), where circled groups 1–3 denote subclusters corresponding to Yunnan wild litchi populations along the Yuanjiang and Xijiang Rivers; and (F) highly homozygous SNPs (observed heterozygosity < 0.4). In (C–F), point shapes indicate germplasm types (squares: ancient trees; triangles: wild accessions; circles: cultivars), while fill patterns correspond to geographic origin (for wild and ancient accessions) or maturation period (for cultivars).

Population structure analysis, founded on unlinked common SNPs, verified this clustering pattern. Throughout the range of *K* = 2–5, Clusters 1 and 2 demonstrated evidently distinct genetic constituents. Ancient trees originating from Sichuan Province persistently presented homogeneous genetic backgrounds that were consistent with YNW. In contrast, the five hybrid clusters (H1–H5) manifested admixed genetic backgrounds (Fig. 2B). Cluster H1 included EMCs and several ancient litchi accessions from Guangdong and Sichuan. Cluster H2 consisted of ancient trees from Fujian, with ‘Songli’ and ‘Songxiang’ as distinct subclusters. Most Fujian accessions resembled ‘Songxiang’ genetically. Cluster H3 contained one EMC and one ancient litchi accession from Sichuan Province. Cluster H4 was predominantly composed of ancient litchi accessions, the cultivar ‘Heiye’ from Guangdong Province, and several accessions from Fujian Province (ZP3, ZP4, and ZP5). Cluster H5 encompassed ancient litchi accessions from Guangdong Province, along with both LMCs and EMCs (Fig. 2A).

Population history analyses frequently focus on common variants; nonetheless, rare variants may provide heightened sensitivity to fine-scale population structure patterns [17, 18]. To explore this, we performed PCA using distinct SNP categories. Common and homozygous SNPs partitioned accessions into two primary clusters with a small number of intermediates, which was consistent with the phylogenetic tree (Fig. 2C and F). Rare SNPs revealed additional structure: PC1 identified two Thai cultivars (‘KALOKA’ and ‘KOM’) as outliers. When the two Thai cultivars were excluded, the sample distribution along PC2 indicated a distinct gradient in the abundance of rare SNPs, with Hainan showing a higher prevalence of rare variants (Fig. 2D; Table S4). PCA utilizing heterozygous SNPs also distinguished two main clusters and hybrids along PC2, while PC1 further divided Cluster 1 into three subgroups corresponding to wild litchi populations distributed along the Yuanjiang and Xijiang Rivers (Fig. 2E). The first subgroup was composed of wild resources from the upstream Yuanjiang River (Yunnan) and Daxin County, Guangxi (GXDXWs). The second subgroup consisted of wild accessions from Vietnam, GXDXWs, and EEMCs. The third subgroup included wild resources from southern Yunnan, ancient litchi trees from Sichuan, and EEMCs. These findings imply that rare and heterozygous variants improve the resolution for uncovering fine-scale population structure in litchi.

### Runs of homozygosity reveal shared genomic segments and historical connectivity

To conduct a more in-depth exploration of the shared ancestry among litchi accessions, an analysis of ROH across all accessions was carried out. A total of 311 484 ROHs were identified, which were then integrated into 8568 distinct ROH clusters. The cumulative genomic length covered by ROHs per individual varied significantly among accessions (Fig. 3A). Notably, 73.81% of the accessions in Cluster 1 fell within the top quartile (>75th percentile) of total individual ROH length across the population. PCA based on the pairwise ROH-sharing matrix (Fig. 3B) revealed distinct population structuring. PC1 effectively differentiated Cluster 1 from Cluster 2 and the hybrid groups. Within Cluster 1, three distinct subgroups were clearly distinguishable: wild accessions originating from Yunnan (top-right), those from the Yunnan–Sichuan region (center), and a cluster encompassing Guangxi–Vietnam along with Hainan wild accessions. The ROH-sharing patterns demonstrated that 90.91% of the ROH clusters were concentrated within Cluster 1, which is in line with an origin within this cluster followed by subsequent dissemination. Interestingly, the genetically discrete wild Guangxi accessions, GXDXWs (Cluster 1) and GXBBWs (Cluster 2), cluster together in the ROH analysis (Fig. 3B), with GXDXWs sharing 44.51% of their ROHs with GXBBWs, highlighting historical connectivity despite genome-wide differentiation.

**Figure 3:**
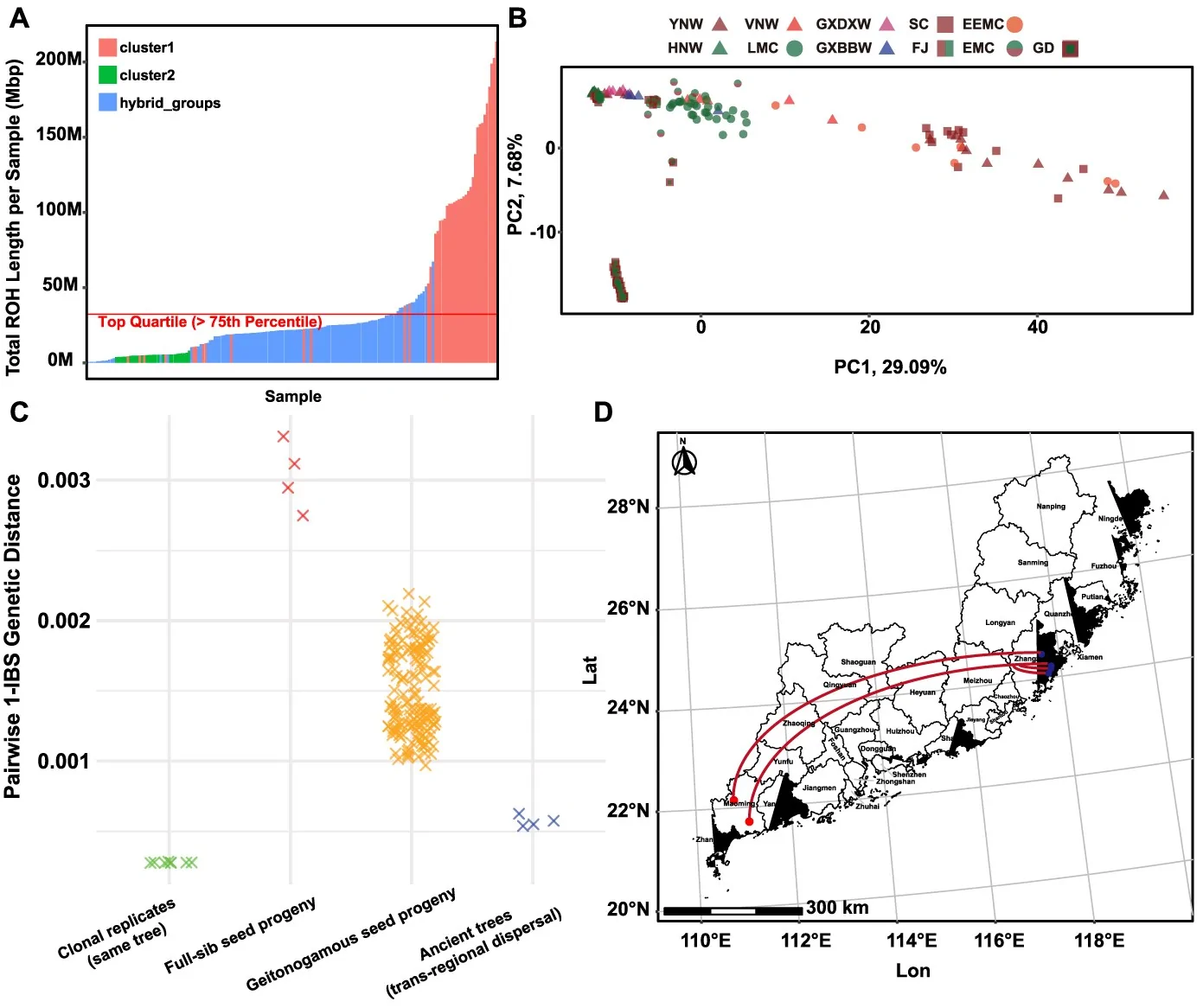
ROH and genetic distances among litchi accessions. (A) Total genomic length of ROH per accession. (B) PCA based on the pairwise ROH-sharing matrix. Point shapes indicate germplasm types: squares for ancient trees; triangles for wild accessions (including Yunnan [YNW], Vietnam [VNW], Daxin, Guangxi [GXDXW], Hainan [HNW], and Bobai, Guangxi [GXBBW]); and circles for cultivars (including extremely early- [EEMC], early- [EMC], and late-maturing [LMC] cultivars), with geographic origin (for wild and ancient accessions) or maturation period (for cultivars) indicated as shown in the legend. (C) Genetic distances among litchi accessions across distinct relationship categories. (D) Geographic distribution of ancient litchi accessions in Guangdong and Fujian exhibiting exceptionally low genetic distances.

### Genetic distances reveal ancient dispersal routes

To elucidate the dispersal history of ancient litchi trees, pairwise identity-by-state (IBS) genetic distances among all sampled individuals were calculated. Clonal replicates (distinct branches from the same tree), geitonogamous progeny, and full-sib progeny from controlled crosses were employed as references (Table S6). The IBS values across the collection exhibited a wide range, mirroring substantial variation in relatedness. The maximum distances (>0.50) were observed between accessions from Yunnan and Hainan, which was in line with pronounced regional differentiation (Table S6). Among the ancient trees in Sichuan and the samples from Yunnan, YB1 (collected in Yibin, Sichuan) and YNW01 (collected in Pingbian, Yunnan) presented the minimum pairwise distance (0.0028), approximating that of full-sib progeny and implying inter-regional dispersal. Remarkably, geographically distant trees, including Jinfeng (JF, collected in Gaozhou, Guangdong Province), SHHY2 (collected in Dianbai, Guangdong Province), and accessions from Longhai (LH1) and Zhangpu in Fujian Province (ZP3, ZP4, and ZP5), demonstrated genetic distances lower than those of geitonogamous progeny and approaching the levels detected for vegetative clones from the same mother plant (average 0.00028) (Fig. 3C and D). Furthermore, ‘Lanzhu’, a cultivar extensively cultivated across Guangdong and Fujian provinces, had a genetic distance of 0.00035 relative to JF, indicating that the two cultivars are clonal variants. These patterns imply human-mediated clonal propagation as a mechanism for the extensive distribution of ancient trees with identical genotypes.

### Species distribution modeling of litchi across climatic periods

In order to reconstruct the historical distribution dynamics of litchi, suitable habitat ranges for the present, the Last Interglacial (LIG; 200–20 ka), and the Holocene (11.7–0.3 ka) were simulated via a Random Forest algorithm based on dimensionally reduced bioclimatic variables. A total of 27 620 raw litchi distribution records were acquired, of which 27 462 were sourced from ancient litchi trees. Within each grid cell, only one occurrence record was retained. Consequently, 1349 unique distribution points were incorporated into the final model construction (Fig. 4A; Table S8). The predicted modern distribution exhibited a close match with the species’ known natural range, suggesting high model accuracy (Fig. 4A and B). During the LIG, highly suitable habitats were confined to present-day Hainan, with additional fragmented areas of moderate suitability distributed along the Yunnan–Vietnam–Guangxi border region, the Indochinese Peninsula, and the eastern coastal zone of China (Fig. 4C). At the LGM, no highly suitable habitats were present within the study area, and only small patches of moderate suitability persisted on the exposed eastern shelf of Hainan and the Indochinese Peninsula (Fig. 4D). From 12.9 to 11.7 ka BP (kilo annum before present), the extent of moderately to highly suitable habitats gradually increased, exceeding that of the LIG and concentrating primarily in Hainan (Fig. 4E and F). During the early- and mid-Holocene, suitable habitats for litchi expanded further into Guangdong, Guangxi, and their adjacent regions. In the late Holocene, scattered patches of suitable habitats emerged in the Yunnan–Vietnam border area (Fig. 4G–I). Generally speaking, the suitable habitats for litchi contracted remarkably during the LGM and then expanded during the Holocene, especially across South China.

**Figure 4:**
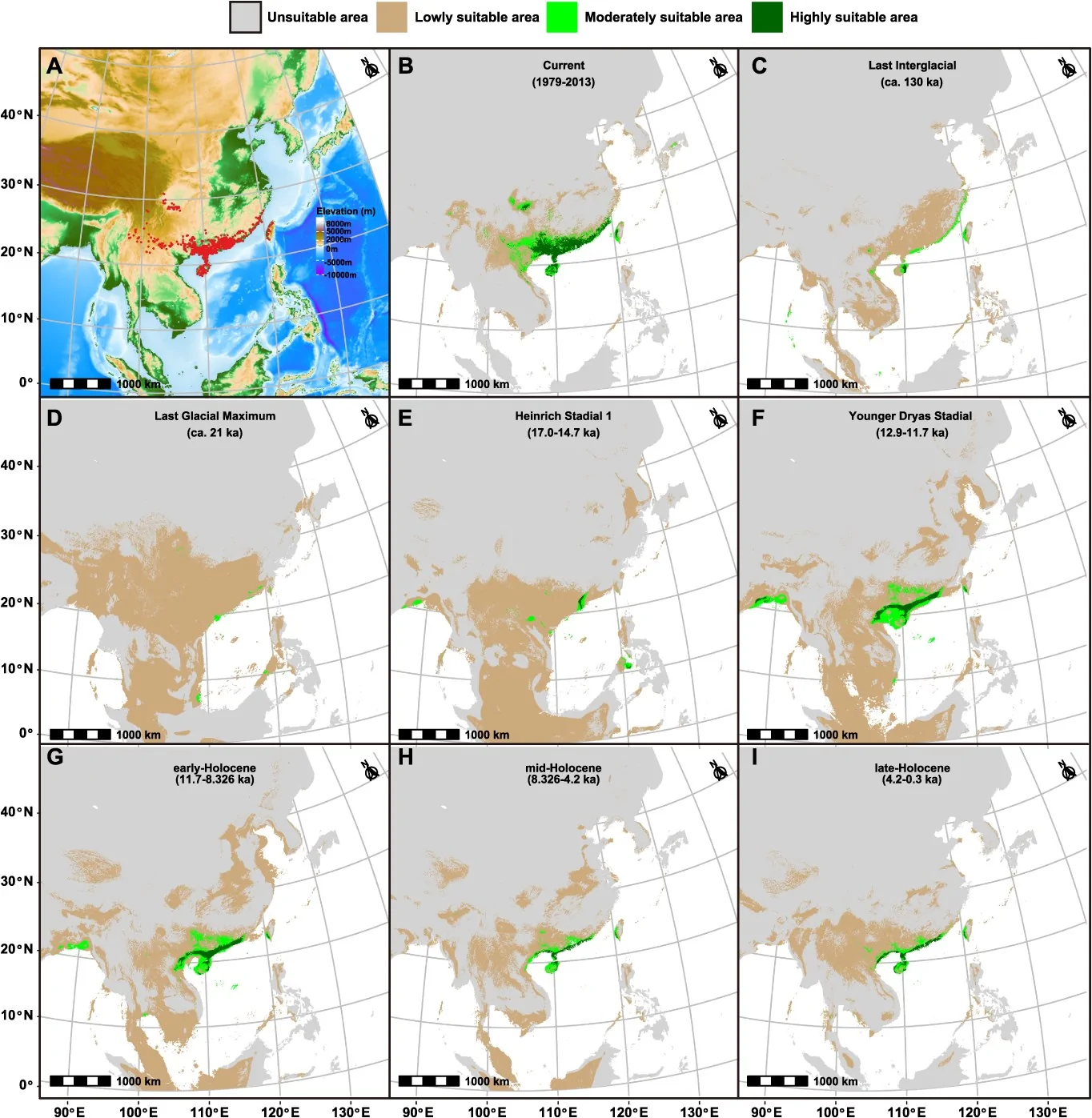
The predicted potential distribution of litchi. (A) Litchi occurrence for SDM. Potential distribution of litchi in China and adjacent regions predicted by the random forest model under (B) current climate conditions, (C) LIG, (D) LGM, (E) Heinrich Stadial 1, (F) Younger Dryas Stadial, (G) early-Holocene, (H) mid-Holocene and (I) late-Holocene.

### Selection signals during litchi domestication

Considering that EEMC and LMC originated independently from the YNW and HNW populations, respectively [1], the subsequent step involves characterizing the selection signatures specific to each domestication event. During the transition from YNW to EEMC, genes associated with defense, development, and stress response were identified within selective sweeps (Fig. 5A). Notably, defense-related genes, including *Wall-associated receptor kinase-like gene (WAKLs*), *Leucine-rich repeat protein 1* (*LRR1*), and *NON-RACE-SPECIFIC DISEASE RESISTANCE 13* (*NHL13*) were under strong selection. Additionally, genes involved in developmental processes and nutrient transport, including *BLISTER* (*BLI*), *WRKY72*, *MYB121*, and *AGAMOUS-LIKE4*(*AGL4*), were also targeted by selection. In the second domestication trajectory, selection signals were also concentrated in regions related to defense, reproduction, and metabolic regulation (Fig. 5B). Gene ontology (GO) enrichment analysis of genes located in selective sweeps showed significant enrichment in aromatic amino acid biosynthesis pathways (Table S9). Among them, *UDP-glycosyltransferase 74F2* (*UGT74F2*), a key enzyme for *salicylic acid glucoside* (*SAG*) formation, emerged as a notable fruit defense candidate. Other candidate genes under selection included *probable pyridoxal 5′-phosphate synthase 1* (*PDX1*), rapid alkalinization factor like-20 (*RALFL20*), and *polygalacturonases* (*PGs*), which are associated with defense responses, as well as *inositol polyphosphate 5-phosphatase* (*5ptase13*) and *ABERRANT POLLEN DEVELOPMENT 2* (*APD2*), which are related to pollen development.

**Figure 5:**
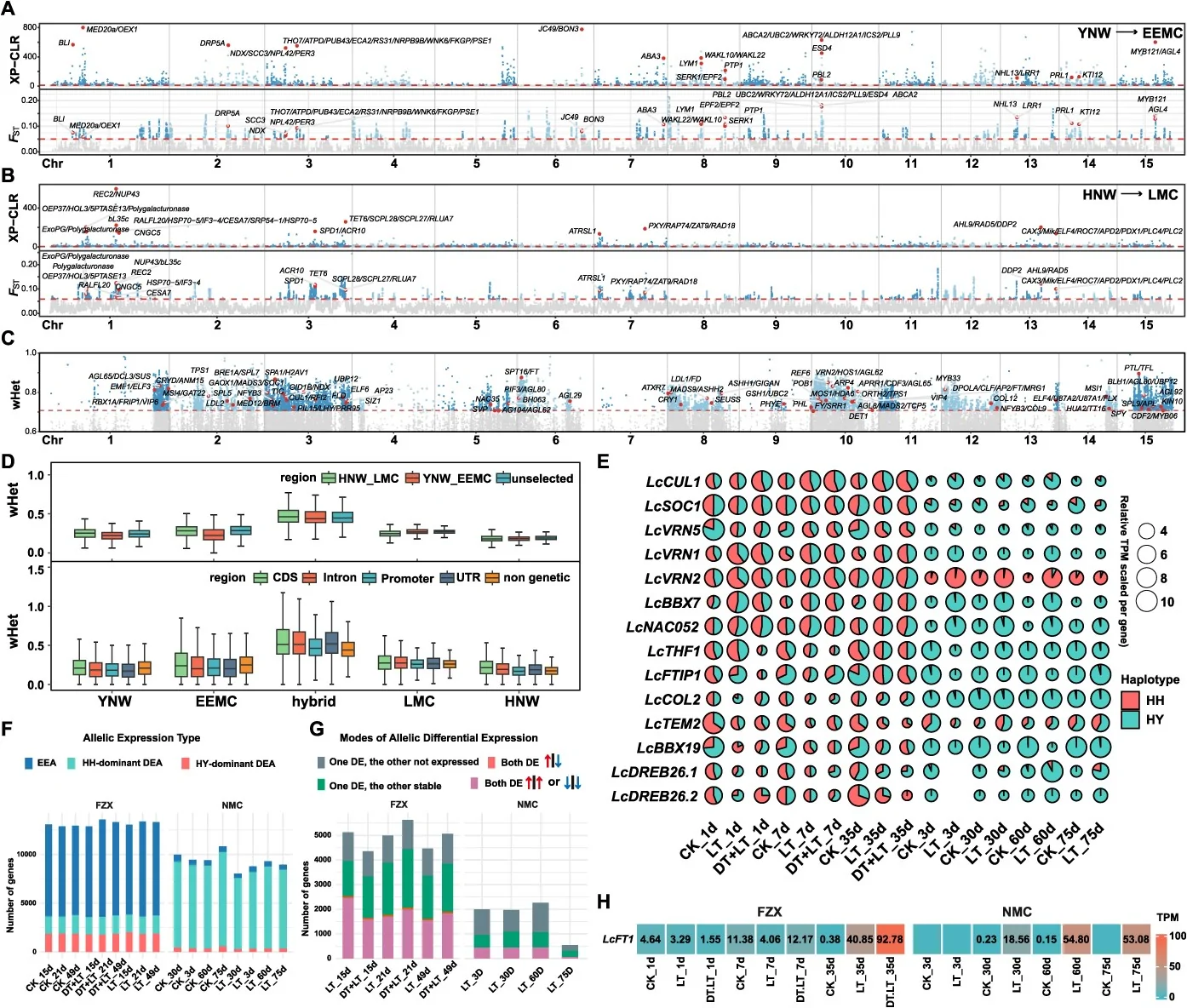
Genomic differentiation, heterozygosity, and ASE in litchi. (A–C) Genome-wide distribution of selective sweeps detected by XP-CLR and FST analysis for YNW vs. EEMC (A) and HNW vs. LMC (B), alongside window-level heterozygosity (wHet) distributions in hybrid groups (C). The horizontal black line indicates the significance threshold (top 5%). (D) Comparison of wHet between selected (YNW/EEMC, HNW/LMC) and nonselected genomic regions across litchi subgroups; wHet across functional genomic elements in different subgroups. (F and G) Genome-wide patterns of allelic expression; (F) Overall allelic expression patterns and (G) classification of alleles into co-regulated and differentially regulated categories. ‘DE’ denotes differentially expressed; upward and downward arrows indicate upregulation and downregulation, respectively. (E and H) Heatmap visualization of flowering-associated genes in ‘FZX’ and ‘NMC’ under floral induction treatments. DT + LT: drought treatment followed by low temperature; LT, low temperature.

Collectively, these findings suggest that the domestication of litchi involved intense and recurrent selection on genes governing defense signaling, reproductive processes, and metabolic pathways, potentially mirroring adaptations to human preferences and cultivation environments.

### Heterozygous blocks and allele-specific expression drive cultivar-specific flowering

In line with the robust yet predominantly lineage-specific selection documented previously, merely 1.6 Mb of the selection sweeps regions were common between the two domestication events. Hybrid accessions demonstrated heightened window-level heterozygosity (wHet) values, nearing 0.5 in both selected and unselected regions (Fig. 5D). When scrutinizing heterozygosity across genomic features, the hybrid group consistently displayed higher wHet in gene regions compared to nongenic regions, a pattern not discerned in the other groups (Fig. 5D). These results imply that heterozygous regions might possess functional importance in hybrid accessions.

Genome-wide analysis indicated the existence of 3404 highly heterozygous blocks (with lengths ranging from 5 to 90 kb and a total length of 32.6 Mb) within hybrid groups, which included 4456 genes. Among these genes, 123 were flowering-related genes (Table S5), showing a significant enrichment in comparison to the genome-wide average (χ^2^ test, *P* < 2.94 × 10^−13^). These blocks harbored major flowering regulators, such as *FLOWERING LOCUS T* (*FT*), *EARLY FLOWERING 4* (*ELF4*), *SQUAMOSA PROMOTER BINDING PROTEIN-LIKE* (*SPL*), *SHORT VEGETATIVE PHASE* (*SVP*), *CONSTANS*-like (*COL*), as well as members of the NAC and DREB families (Fig. 5C). Consistent with their mosaic genomes, hybrid accessions exhibited flowering times and chilling requirements that were intermediate between those of the EEMC and LMC.

To assess whether genomic heterozygosity is manifested in regulatory divergence, allele -specific expression analysis was performed on the early-flowering cultivar ‘FZX’ (Cluster 5, Fig. 3A and B) and the late-flowering cultivar ‘Nuomici’ (‘NMC’) (Cluster 4, Fig. 3A and B) under floral induction treatments. In ‘FZX’, haplotypes were primarily equivalently expressed alleles (EEAs) across different conditions. Conversely, ‘NMC’ demonstrated a pronounced haplotype bias, with expression frequently being dominated by the HNW-derived haplotype and a significantly lower number of EEAs (Fig. 5F).

A comparative analysis of floral induction and control conditions identified four allelic regulation patterns: (1) one allele was differentially expressed while the other was unexpressed, (2) one allele was differentially expressed while the other remained stable, (3) both alleles were differentially expressed in opposite directions, and (4) both alleles were differentially expressed in the same direction (Fig. 5G). In ‘FZX’, patterns (1) and (2) were the predominant ones, while coordinated regulation (pattern 4) was also relatively frequent, and opposite regulation (pattern 3) occurred only infrequently. In ‘NMC’, pattern (1) was overwhelmingly dominant, whereas patterns (2) and (4) were less prevalent, and pattern (3) was almost nonexistent (Fig. 5G). These results imply that the observed haplotype divergence might serve as the genetic foundation for the distinct flowering times among litchi cultivars.

Allelically modulated genes showed clear cultivar- and haplotype-specific patterns (Fig. 5H). In ‘FZX’, flowering promoters *Cullin1*(*CUL1*), *VERNALIZATION 1*(*VRN1*), and *SUPPRESSOR OF OVEREXPRESSION OF CO1*(*SOC1*) displayed balanced haplotype expression and were slightly induced at 15°C. In ‘NMC’, expression was almost exclusively from the HH haplotype; *LcCUL1* abundance was lower than in ‘FZX’, and *LcSOC1* declined. *LcVRN5* in ‘FZX’ decreased overall, yet the HY haplotype remained upregulated under low temperatures. In ‘NMC’, only HH was expressed and showed little response. Among repressors, *LcVRN2* in ‘FZX’ was induced from the HY haplotype only, whereas ‘NMC’ expressed HY alone with stronger and faster induction. Negative regulators *B-box 7*(*BBX7*), NAC domain containing protein 52 (NAC052), and *LcCOL2* were HH-specific in ‘NMC’ and accumulated to higher levels than in ‘FZX’ after a cold exposure.

FT-related components also diverged (Fig. 5H). *LcFT1* increased in both cultivars (Fig. 5I). *THYLAKOID FORMATION 1* (*THF1*), which sequesters FT at the chloroplast membrane, was continuously downregulated in ‘FZX’ during floral induction, especially under combined low temperature and predrought, consistent with increased free FT and earlier flowering. In ‘NMC’, only HH was expressed and the reduction was transient at early induction. *LcFTIP1* showed haplotype-specific regulation: in ‘FZX’, HY increased and HH decreased; in ‘NMC’, only HH was expressed and remained below control for most of the induction period. *LcTEM2* exhibited opposite haplotype biases: HY predominated and declined steadily in ‘FZX’, whereas HH predominated in ‘NMC’ with only a brief early reduction. *LcBBX19* declined rapidly in ‘FZX’ under cold, while ‘NMC’ required greater chilling accumulation to reach comparable reductions. Together, these results indicate that heterozygosity at flowering loci and allelic imbalance shape the divergent flowering responses of litchi cultivars.

### Heterozygosity and haplotype-resolved expression shape resistance in ‘Heiye’

The cultivar ‘Heiye’ demonstrates resistance to *Peronophythora litchii* and represents a core genetic lineage within ancient litchi populations, which encompasses the oldest existing tree. To clarify the genetic foundation of resistance in ‘Heiye’, a genome-wide scan of genotypic diversity was carried out by comparing ancient ‘Heiye’ accessions (Table S1) with other hybrids. By ranking genome windows based on the disparity in Shannon genotypic diversity, the top 5% were discovered to be enriched with genes associated with immune regulation, hypersensitive response, programmed cell death, and defense-related cell-wall modification (Fig. 6C). These regions harbored resistance-related homologs, including *aspartic proteases 1* (*LcAP1*), *Respiratory burst oxidase homolog protein B* (*RBOHB*), and *Regulator of CBF gene expression 3* (*RCF3*) (Fig. 6A).

**Figure 6:**
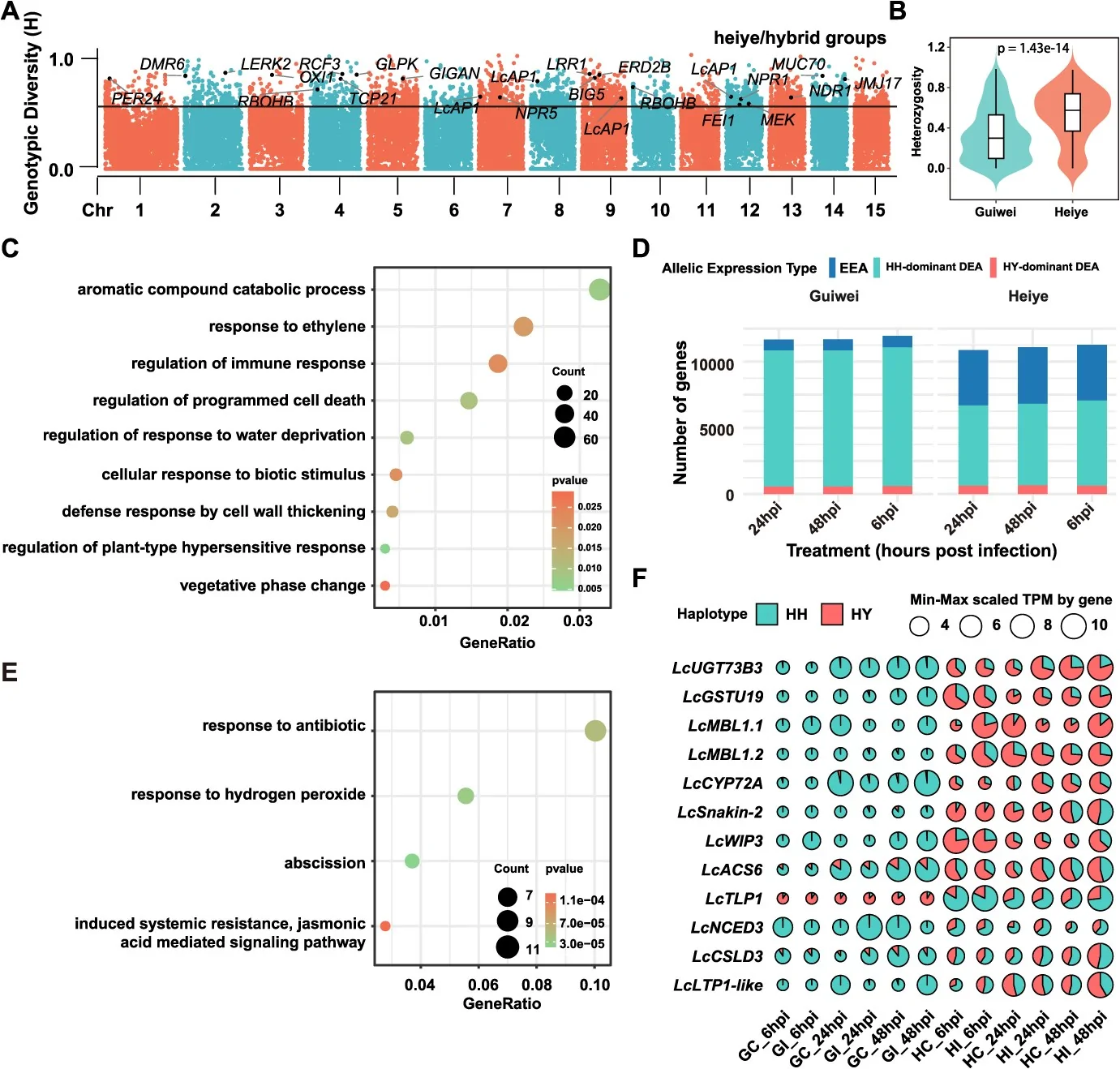
The genomic diversity and ASE underlying disease resistance in litchi: comparative analyses of ‘Heiye’ and ‘Guiwei’ during *P. litchii* infection. (A) Difference in genome-wide genotypic Shannon diversity of ancient litchi population (‘Heiye’) and other hybrids, the horizontal black line indicates the significance threshold (top 5%). (B) Gene-region heterozygosity in defense-related genes of ‘Heiye’ vs. ‘Guiwei’ (*t*-test). (C) GO enrichment of genes located within the top 5% genomic regions exhibiting elevated Shannon diversity ratios between ancient litchi population (‘Heiye’) and other hybrids. (D) Differential allelic expression patterns and heatmap of allelic expression of resistance-associated genes. (E) GO enrichment of genes exhibiting differential allelic dominance between ‘Heiye’ and ‘Guiwei’ after *P. litchii* infection. (F) in pericarp of ‘Heiye’ and ‘Guiwei’ after *P. litchii* Infection (C, control conditions; G, ‘Guiwei’; H, ‘Heiye’; hpi, hours postinoculation; I, inoculated).

Previous research identified nineteen defense-related genes that are upregulated in the resistant ‘Heiye’ compared to the susceptible ‘Guiwei’ (‘GW’) upon *P. litchii* infection [19]. These include key genes involved in lignin biosynthesis, such as cinnamic acid 4-hydroxylase (*C4H*) and peroxidase (*POD*), as well as reactive oxygen species (ROS) scavenging enzymes like superoxide dismutase (*SOD*) and catalase (*CAT*). Whole-genome resequencing indicated that ‘Heiye’ has an admixed genetic background from Cluster 1 and Cluster 2, while ‘GW’ is predominantly homogeneous from Cluster 2 (Fig. 2A and B). Subsequently, the gene-region heterozygosity of these nineteen genes was examined. In comparison with ‘GW’, ‘Heiye’ demonstrated significantly higher heterozygosity at these loci (*t*-test, *P* = 1.43 × 10^−14^; Fig. 6B), suggesting a correlation between allelic diversity and enhanced disease resistance.

Utilizing published transcriptomic data [20], haplotype-resolved expression analysis was conducted to investigate allele-specific transcription in ‘Heiye’ and ‘GW’ at 6, 24, and 48 hours postinoculation. In ‘GW’, over 87.81% of allele expression was HH-dominant, with only ~7% of EEAs . Conversely, ‘Heiye’ exhibited a notably reduced HH-dominance (~56.14%) and a five-fold higher proportion of EEAs (about 37.95%) across all time points (Fig. 6D). In total, 218 genes displayed distinct allelic dominance between the two cultivars after infection. GO enrichment revealed significant associations with abscission, response to hydrogen peroxide, jasmonic acid–mediated induced systemic resistance, and antibiotic response (Fig. 6E).

In ‘Heiye’, multiple defense-associated genes demonstrated active expression of both HH and HY haplotypes, accompanied by allelic imbalance. In contrast, in ‘GW’, the expression was predominantly confined to a single haplotype at a diminished level. For instance, glycosyltransferases (*UGT73B3*), *Glutathione S-transferase U19*(*GSTU19*), *mannose-binding lectins* (*MBLs*), *Cytochrome P450*(*CYP72A*), *Snakin-2* (*SN2*), *WPP domain-interacting protein 3* (*WIP3*), and *ACC Synthase 6* (*ACS6*) were HY-dominant differentially expressed alleles (DEAs) in ‘Heiye’ and were upregulated subsequent to infection by *P. litchii.* Conversely, in ‘GW’, the corresponding HH haplotypes exhibited minimal alteration or were suppressed. *Thaumatin-like 1* (*TLP1*) was HH-dominant in ‘Heiye’ and was strongly induced at 48 hours postinoculation (hpi), whereas in ‘GW’, the HY haplotype was expressed but remained stable. *Cellulose synthase like* (*CSLD3*) and *litchi lipid transfer protein 1-like* (*LcLTP1-like*) were equivalently expressed in ‘Heiye’, with the HY haplotypes responding to the infection. However, in ‘GW’, the HH haplotypes showed no induction or were suppressed (Fig. 6F). These results suggest that ‘Heiye’ harbors greater allelic diversity and balanced haplotype expression, and key defense-associated genes contribute to its resistance against *P. litchii*.

## Discussion

Despite a cultivation history spanning over two millennia in Southern China, a comprehensive whole-genome resequencing of ancient litchi resources has remained elusive until now. In this study, we present a high-quality variation dataset derived from 175 accessions, including 93 *in situ* ancient trees. By integrating genomic evidence with paleoclimate modeling, we reconstruct a definitive evolutionary trajectory for litchi: originating in Yunnan, surviving the LGM in Hainan refugia, and undergoing an asymmetric postglacial expansion followed by human-mediated dispersal and domestication.

Divergence time estimations suggest that the divergence between litchi and longan occurred ~10.1 million years ago [1]. Fossil-based climate reconstructions from Yunnan during the late Miocene (11.62–5.33 Ma) indicate that the region offered suitable habitats for litchi during that era [21–24]. Population history analyses employing SMC++ suggest that the divergence between the Hainan (HNW) and Yunnan (YNW) wild populations took place around 18 000 years ago [1]; however, the underlying drivers of this divergence have not been fully explored. Our findings disclose that during the LIG, highly suitable habitats for litchi were confined to present-day Hainan. At the LGM, suitable habitats contracted significantly toward lower latitudes, with only small areas of moderate suitability remaining on the exposed eastern shelf of Hainan and the Indochinese Peninsula (Fig. 4). This probably contributed to the spread of litchi from its origin center to Hainan, over 1000 km away, resulting in genomic and ecological adaptation divergence. Pollen records, megafossil evidence, and SDM all corroborate the hypothesis of a cooler and drier climate in southern China during this period. In Maoming city, an area currently abundant in ancient litchi trees, cold-tolerant species such as *Pinus armandii* were widespread [25, 26]. After the LGM, the recovery of suitable habitats around Yunnan was slower compared to those in Hainan and southern China. Consistent with this pattern, the effective population size (*Ne*) of HNW showed an initial decline followed by a rapid increase, whereas YNW exhibited a more gradual recovery and experienced a more pronounced bottleneck [1]. This divergence in population dynamics is presumably attributable to variations in the rate of habitat restoration, highlighting the role of climate in shaping lineage demography and subsequent range dynamics.

Broadly, genomic data discern two principal wild litchi progenitors originating from Yunnan and Hainan, alongside several admixed clusters [1, 12, 13]. Building upon this framework, a major contribution of this study is the resolution of fine-scale genetic structures that were previously overlooked. By analyzing rare variants, we uncovered a cryptic level of population stratification and identified an exceptionally high frequency of rare alleles in Hainan (Fig. 2D). This fine-scale resolution reaffirms Hainan’s status as a primary genetic reservoir and a critical glacial refugium, where rare variants have accumulated through prolonged isolation. Furthermore, the use of heterozygous SNPs allowed us to partition the Yunnan-associated lineage into three distinct subgroups distributed along the Yuanjiang and Xijiang Rivers (Fig. 2E). This aligns with recent liquid-chip results [13], solidifying the model of fine-scale subdivision in Yunnan. The concentration of over 90% of ROH within Yunnan lineages is consistent with genomic signatures of historical bottlenecks expected at a species’ center-of-origin. Furthermore, the sharing of long ROHs between the genetically discrete yet geographically close GXDXW and GXBBW groups offers new insights into regional connectivity, supporting the hypothesis that a historical genetic corridor through Guangxi may have shaped the interactions between Yunnan and Hainan wild lineages.

Human agency then reshaped distribution and cultivation of litchi. Genomic evidence indicates that ancient Sichuan litchi share genetic traits with wild populations from Yunnan (Fig. 2A and B). Accordingly, ancient Chinese literature records that prior to 316 BCE, the Bo people in Sichuan carried out large-scale litchi cultivation [2]. The fragmented microclimates limited the suitability of litchi cultivation in both provinces. Nevertheless, the connection between Yunnan and Sichuan—as evidenced by archaeological, textual, and historical records dating back to at least the late second millennium BCE [27]—validates an early human-mediated introduction of litchi from Yunnan to Sichuan. Most ancient litchi trees in Guangdong and Fujian, along with some in Sichuan, exhibited mixed genetic backgrounds typical of cultivated varieties. A subset of ancient trees in Guangdong clustered with wild accessions from Hainan, and phylogenetic analyses verified extensive germplasm exchange among these regions. The extremely low genetic distances among ancient trees in Fujian and Guangdong—lower than those in geitonogamous progeny and approaching those of single-mother vegetative clones (Fig. 3C and D)—imply a shared clonal origin rather than sexual reproduction. This pattern offers rare direct evidence of historical cross-regional germplasm exchange facilitated by vegetative propagation. These findings are consistent with the Zhangzhoufuzhi (1776), which recorded the introduction of ‘Lanzhu’ from Guangdong into Zhangpu and Zhangzhou. Collectively, these results illustrate that the dissemination and long-term conservation of superior litchi germplasm across regions were accomplished through centuries of clonal propagation.

Taking into account the independent domestication origins of EEMCs and LMCs from distinct wild gene pools [1], our findings suggest that each lineage underwent substantial yet predominantly lineage-specific selection on comparable functional pathways. The minimal overlap in selection signals validates their genetic distinctiveness. In both lineages, selection was concentrated on genes associated with defense, development, and metabolism. Cultivars with admixed genetic backgrounds exhibited highly heterozygous genomic regions, which were enriched with crucial flowering regulators (Fig. 5C), consistent with their intermediate flowering times and chilling requirements when compared to the EEMC and LMC groups.

Moreover, we established a mechanistic link between heterozygosity, haplotype-biased regulation, and the coordination of key agronomic traits, specifically flowering time and disease resistance. During floral induction, the early-flowering ‘FZX’ and late-flowering ‘NMC’ demonstrated ASE divergence in several flowering-time regulatory factors. These factors include genes previously reported in litchi or homologous genes characterized in other plant species (Fig. 5F–I). The positive regulatory factors encompass *LcCUL1*, *LcVRN1*, *LcVRN5*, *LcFTIP1*, and *LcSOC1* [28–31], whereas the negative regulatory factors include *LcBBX7*, *LcNAC052*, *LcCOL2*, *LcTHF1*, *LcTEM2*, and *LcBBX19* [32–39].

Parallel patterns of disease resistance against *P. litchii* were identified between the resistant cultivar ‘Heiye’ and the susceptible cultivar ‘GW’. Haplotype-resolved transcriptomic analysis indicated that in ‘Heiye’, defense-associated genes exhibited biallelic expression with allelic imbalance, whereas in ‘GW’, the expression was predominantly monoallelic and at a relatively lower level (Fig. 6E and F). Key defense-related genes previously reported in litchi or their homologs characterized in other plant species—including *LcUGT73B3*, *LcGSTU19*, *LcMBLs*, *LcCYP72A*, *LcSnakin-2*, *LcWIP3*, *LcACS6*, *LcTLP1*, *LcCSLD3*, and *LcLTP1-like*—were recognized [40–49] exhibited cultivar-specific haplotypic expression patterns, with a significant induction upon infection in ‘Heiye’, whereas it mostly remained unresponsive or was repressed in ‘GW’. Collectively, these findings underscore the significance of preserving heterozygosity and haplotype-specific regulatory variation at crucial loci for optimizing complex traits, such as flowering time and disease resistance, in litchi.

Our research integrates ancient-tree genomics, paleoclimate modeling, and haplotype-resolved transcriptomics to reconstruct the sequence of origin, refugium, and re-expansion, quantify human-mediated movement, and elucidate the underlying genetic basis of key traits. The limitations of this study include a restricted sampling range and sample size, which may not fully capture the breadth of genetic diversity within ancient litchi populations. Furthermore, long-term *in situ* observations of agronomic traits in ancient trees remain limited. To address these gaps, future investigations should broaden the sampling scope across Mainland Southeast Asia, the Leizhou Peninsula, and Hainan Island. Increasing sample sizes and incorporating multiyear phenotypic assessments will be critical. Additionally, haplotype-level association studies and functional validation of key genes through targeted editing or expression assays are essential to deepen our understanding.

## Materials and methods

### Sample collection and sequencing

Samples were collected from 107 litchi trees across China (Table S1). Fresh leaves were obtained from all samples, flash-frozen in liquid nitrogen, and then stored at −80°C for DNA extraction. Sample quality evaluation, library quality control, and sequencing were carried out in accordance with the standardized protocols of Illumina Inc. DNA was extracted via the cetyltrimethylammonium bromide (CTAB) method. DNA samples, verified for concentration, purity, and integrity, were utilized to construct libraries with an approximate insert size of 350 bp using the NEB DNA Library Rapid Prep Kit. Sequencing of the 150-bp paired ends was executed on an Illumina Novaseq6000.

To transform the raw data into a clean dataset, Cutadapt (v1.9.0) was utilized for adapter removal [50]. Reads containing over 10% unknown nucleotides (N) or those with more than half of their bases having a quality value below 10 were discarded using FASTP [51].

### Alignment and variant calling

High-quality clean reads were aligned to the reference genome of the litchi cultivar ‘Feizixiao’ with the utilization of BWA software (version 0.7.17) [52]. PCR duplicates were eliminated through the application of the Picard MarkDuplicates tool (version 2.25.1). Population-level variant detection was carried out using the Genome Analysis Toolkit HaplotypeCaller (GATK, version 4.2.0). SNPs that satisfied the recommended ‘hard-filter’ criteria (QD < 2.0, FS > 60.0, MQ < 40.0, MQRankSum < −12.5, ReadPosRankSum < −8.0) were retained for subsequent analysis [53].

SNPs selected for annotation were acquired subsequent to comprehensive filtration using PLINK (v1.90) and bcftools 1.13 [54, 55]. The selection criteria entailed retaining only biallelic SNPs within a depth range from one-third to two times the average genome depth per sample. Variants characterized by a genotyping rate lower than 90%, a MAF < 5%, and a quality score below 30 (-vcf-min-qual 30) were excluded. The resultant subset of high-quality SNPs was annotated via Annovar [56]. The genomic structure and variation information were visualized utilizing the circlize R package [57].

### Phylogeny construction and population structure analysis

PCA, population structure analysis, and phylogenetic analysis were carried out to explore the overall genetic background of the samples. Regarding the PCA, biallelic SNPs within one-third to two times the average genome depth per sample were classified into four groups: common SNPs (MAF ≥ 5%), rare SNPs (MAF < 5%), highly heterozygous SNPs (observed heterozygosity > 0.8), and homozygous SNPs (observed heterozygosity < 0.4). The PCA was executed using PLINK (v1.90) [54] on these SNP sets, and scatter plots were produced using the ggplot2 R package [58].

To investigate genetic ancestry, the ADMIXTURE software (version 1.3) [59] was employed. SNPs with a missing genotype rate surpassing 40% or those in linkage disequilibrium were excluded. Unlinked SNPs were selected using PLINK (version 1.90), with a sliding window of 50 SNPs and a step size of 10 SNPs. SNPs with an *R*^2^ value > 0.1 were filtered out, in accordance with the recommendations in the ADMIXTURE user manual. Ancestry bar plots were generated using the Pophelper R package (version 2.2.7) [60]. For the construction of a phylogenetic tree, biallelic SNPs within one-third to two times the average genome depth per sample were retained using PLINK (version 1.90), while those with an MAF below 10% or a missing genotype rate above 40% were excluded. The neighbor-joining method was utilized for tree construction, implemented via the VCF2Dis software [61].

### Runs of homozygosity detection

ROH were identified through the utilization of PLINK v1.9 based on common SNPs. A sliding window encompassing 50 SNPs was implemented, allowing a maximum of one heterozygous genotype and five missing genotypes per window. ROH segments were delineated as regions with a minimum length of 10 kb, containing at least five SNPs. Adjacent ROH segments were amalgamated if the distance between them was no >  100 kb. A genome-wide heterozygosity threshold of 5% was utilized to categorize segments as ROH.

ROH segments with an overlap exceeding 50% of either segment’s length were identified using the bedtools intersect tool. Subsequently, a graph-based method was implemented using the NetworkX Python package. In this approach, each ROH segment was considered a node, and an edge was drawn between two nodes if their corresponding ROH segments satisfied the aforementioned overlap criteria. ROH segments shared by more than three accessions were then classified as ROH clusters.

### Species distribution modeling

Nineteen bioclimatic variables were acquired from PaleoClim (http://www.paleoclim.org) at a resolution of 2.5 arc-minutes. Litchi occurrence records were collated from field investigations, the Global Biodiversity Information Facility (GBIF, https://www.gbif.org), ancient and famous trees information management platforms (Table S7), official archives of ancient trees, and published scholarly works [62]. To alleviate spatial clustering impacts, the records were standardized to one per 2.5 arc-minute grid cell.

The parameters of the Maximum Entropy (Maxent) model were optimized via ENMeval [63]. Subsequently, SDMs were constructed in the R environment using Biomod2, with 10 algorithms employed: Artificial Neural Network, Classification Tree Analysis, Flexible Discriminant Analysis, Generalized Additive Model, Generalized Linear Model, Gradient Boosting Machine, Random Forest, Surface Range Envelope, Multivariate Adaptive Regression Splines, and the optimized Maxent [64]. For all models except Maxent, default settings were applied. The models were trained with 70% of the occurrence data and tested with the remaining 30%, using 10 000 randomly generated pseudo-absences utilized. Each model was replicated 50 times. The performance of the models was assessed using the True Skill Statistic, Cohen’s Kappa coefficient, and the Area Under the Receiver Operating Characteristic Curve. Habitat suitability was classified in accordance with the Intergovernmental Panel on Climate Change thresholds: *P* < 0.05 (unsuitable), 0.05–0.33 (low), 0.33–0.66 (moderate), and ≥0.66 (high).

### IBS calculation

To establish reference values for genetic distances under diverse kinship relationships, a dataset was generated through 60× resequencing of kinship samples. Clonal replicates were exemplified by samples collected from distinct branches of the same tree, encompassing four leaves from HZ400 and two leaves from HZ26. Geitonogamous progeny were represented by 17 geitonogamous offspring of cultivar HML, while full-sib progeny were represented by two offspring from the cross HML × JJHN and three from the cross HML × FSHDL. Variant calling was carried out using GATK GenotypeGVCFs with the ‘all-sites’ option, and only SNPs that passed strict filtering and contained no missing sites were retained. Subsequently, pairwise genetic distances were computed in PLINK v1.90 with the ‘distance 1-ibs square’ option.

### Detection of selective signals

To identify genomic regions correlated with the early-stage domestication of litchi, cross-population composite likelihood ratio (XP-CLR) and fixation index (FST) analyses were carried out between wild and cultivated accessions [55, 65]. Two distinct domestication trajectories were investigated: (i) from wild accessions in Yunnan to EEMCs (Cluster 1) and (ii) from Hainan wild accessions to LMCs (Cluster 2).

In each comparison, variant data were extracted from the rigorously filtered VCF dataset and subsequently refined to retain solely biallelic SNPs with a MAF of at least 0.05 and a missing genotype rate of no more than 40%. XP-CLR scores were calculated using the Python version of xp-clr (https://github.com/hardingnj/xpclr) with the following parameters: the recombination rate (--rrate) was set at 3.7275 × 10^−8^, which was derived from the average marker distance of a genetic linkage map [66]; the linkage disequilibrium threshold (--ld) was set at 0.90; and the maximum number of SNPs per window (--maxsnps) was adjusted according to SNP density (10 000 for comparison 1 and 7000 for comparison 2). For both XP-CLR and FST analysis, the sliding window size was 100 kb with a step size of 10 kb. Candidate selective sweep regions were defined as the intersection of genomic regions ranking in the top 5% for both XP-CLR scores and FST values.

Window-level heterozygosity (wHet) was computed to evaluate genomic heterozygosity. Initially, the observed heterozygosity (OHet) at individual SNP sites was calculated using PLINK v1.90. Subsequently, genome-wide wHet was estimated via a sliding-window method with a window size of 5 kb and a step size of 2.5 kb, by averaging the OHet values across all SNPs within each window. Regarding functional genomic elements, wHet was determined as the mean OHet across the corresponding gene region. In hybrid groups, the top 5% of genomic windows exhibiting the highest wHet values were defined as highly heterozygous blocks.

Considering the admixed genetic background of the cultivar ‘Heiye’ and the potential function of heterozygous alleles and genomic blocks in favorable traits, we quantified the disparities in genome-wide genotypic diversity by employing the Shannon index (*H′*).


$$ {H}^{\prime }=-{\sum}_{i=1}^n{p}_i\ln{p}_i, $$

where *n* is the number of distinct genotypes and *p_i_* is the relative frequency (proportion) of the 𝑖th genotype.

The ancient litchi population of ‘Heiye’ was designated as Group 1, while the hybrid accessions excluding this population were classified as Group 2. For each SNP site, the Shannon diversity index (H′) was calculated independently for both groups. Subsequently, these values were averaged within sliding windows of 5 kb with a step size of 2.5 kb to acquire window-level diversity estimates. For each window, the difference in *H′* between the two groups (Group 2 minus Group 1) was determined, and the top 5% of windows with the largest positive differences were regarded as candidate regions.

Functional annotation of genes situated within the candidate region was accomplished through BLAST searches against the SwissProt database. Genes associated with flowering were identified as the research of Hu *et al*. [1]. Enrichment analyses of GO and Kyoto Encyclopedia of Genes and Genomes (KEGG) pathways were executed using clusterProfiler v4.0 [67].

### ASE analysis

To analyze allelic expression in litchi leaves during floral induction, the expression patterns of EMC ‘FZX’ and LMC ‘NMC’ were compared to their respective controls, using publicly available transcriptome data [68, 69]. For ‘NMC’, the control group (CK, no floral induction treatment) was maintained in a growth chamber at 25/20°C (day/night temperature, 12-hour light/dark cycle) with a relative humidity of 75%–85% and natural light. The flowering induction treatment (L, low temperature) was conducted in a growth chamber at 15/8°C, under identical light and humidity conditions. After 60 days of chilling, the trees were rewarmed to 25/20°C. At the time points of 3, 30, 60, and 75 days (floral initiation), leaves from both groups were sampled. Terminal shoots from three to four trees were pooled as one replicate (three biological replicates for each time point) [68]. For ‘FZX’, prior to low-temperature treatment, all samples (CK, LT, and DT + LT) were grown at room temperature (25 ± 2°C) for 14 days. Drought stress was applied to the DT + LT treatment by adjusting the soil moisture to 25% relative water content, and the relative humidity in the chamber was set to 30% to accelerate water evaporation. At the 15th day, both LT and DT + LT plants were transferred to a low-temperature chamber set at 15°C for 35 days. Following this, the plants were returned to the room temperature chamber and fully watered. Flowering time and flower quantity observations showed no flowering in the CK group. The DT + LT treatment resulted in 100% flowering shoots, which flowered earlier than the LT group (75% flowering shoots). Sequencing libraries were constructed by pooling equal quantities of RNA from three biological replicates, as described by Shen *et al*. [69]. For this study, we analyzed RNA-seq data from samples collected at 15, 21, and 49 days.

To investigate ASE associated with *P. litchii* resistance, we utilized published transcriptomic data from Sun *et al*. [20]. This dataset compares the resistant cultivar ‘Heiye’ against the susceptible cultivar ‘Guiwei’ under *P. litchii* inoculation and mock conditions. RNA-seq libraries were generated from fruit pericarps collected at 6, 24, and 48 hours postinoculation (hpi), with three biological replicates per treatment.

ASE analysis was performed according to the methodology described by Hu *et al*. [1]. To facilitate ASE detection, a composite reference genome was constructed by merging the sequences of the HY and HH haplotypes (denoted as HY-HH). RNA-seq reads were aligned to the HY-HH reference using STAR (2.7.11b) with the following parameters: --alignIntronMax 20 000 --alignMatesGapMax 25 000 --outFilterMultimapNmax 1. Transcript abundance (TPM) and raw read counts were quantified using featureCounts, considering only uniquely mapped reads. Genes were classified into DEAs or equivalent expressed alleles (EEAs) based on the following criteria adapted from Hu *et al*. [1]:

DEAs: Required a minimum read count of ≥10 and a TPM ≥ 1 for at least one allele. Using a threshold of adjusted *P* value <0.05, genes were defined as HY-dominant if the TPM_HY_/TPM_HH_ ratio was ≥2, and HH-dominant if the ratio was ≤0.5.

EEAs: Required both alleles to have a read count ≥ 10 and a TPM ≥ 1, with a TPM_HY_/TPM_HH_ ratio between 0.5 and 2.0.

For datasets lacking biological replicates, differential expression between experimental conditions was calculated based on TPM fold changes using R (version 4.3.2), and |log₂(fold change)| > 1 as the significance thresholds. For datasets with biological replicates, DESeq2 was employed to identify differentially expressed genes (DEGs) and DEAs. DEGs were identified using an adjusted *P* value < 0.05 and |log₂(fold change)| > 1 as the significance thresholds. Genes were then categorized based on their allele-specific differential expression patterns: (i) one allele was differentially expressed while the other was unexpressed (failing to meet the minimum criteria of read count ≥10 and a TPM ≥ 1; (ii) only one allele was differentially expressed, while the other was expressed but showed no significant change; (iii) both alleles were differentially expressed in opposite directions; and (iv) both alleles were differentially expressed in the same direction.

## Supplementary Material

Web_Material_uhag226

## Data Availability

The WGS data used in this study have been deposited in the Genome Sequence Archive (Genomics, Proteomics & Bioinformatics 2025) in National Genomics Data Center (Nucleic Acids Res 2025), China National Center for Bioinformation/Beijing Institute of Genomics, Chinese Academy of Sciences (GSA: CRA031948) that are publicly accessible at https://ngdc.cncb.ac.cn/gsa.
